# Designing and Evaluating a Nutrition Recommender System for Improving Food Security in a Developing Country

**DOI:** 10.34172/aim.2023.93

**Published:** 2023-11-01

**Authors:** Shahabeddin Abhari, Kamran B. Lankarani, Leila Azadbakht, Sharareh R. Niakan Kalhori, Reza Safdari, Sara Emamgholipour Sefiddashti, Ali Garavand, Saeed Barzegari, Sahand Moradi

**Affiliations:** ^1^Department of Health Information Management, School of Allied Medical Sciences, Tehran University of Medical Sciences, Tehran, Iran; ^2^Ubiquitous Health Technology Lab, School of Public Health Sciences, Faculty of Health, University of Waterloo, Waterloo, ON, Canada; ^3^Health Policy Research Center, Institute of Health, Shiraz University of Medical Sciences, Shiraz, Iran; ^4^Department of Community Nutrition, School of Nutritional Sciences and Dietetics, Tehran University of Medical Sciences, Tehran, Iran; ^5^Peter L. Reichertz Institute for Medical Informatics, University of Braunschweig, Institute of Technology and Hannover Medical School, Braunschweig, Germany; ^6^Department of Health Management and Economics, School of Public Health, Tehran University of Medical Sciences, Tehran, Iran; ^7^Department of Health Information Technology, School of Allied Medical Sciences, Lorestan University of Medical Sciences, Khorramabad, Iran; ^8^Department of Paramedicine, Amol School of Paramedical Sciences, Mazandaran University of Medical Sciences, Sari, Iran; ^9^The York Management School, University of York, York, UK

**Keywords:** Food security, Health informatics, Meal, Nutrition, Recommender systems

## Abstract

**Background:**

Due to the increased price of foods in recent years and the diminished food security in Iran, nutrition recommender systems can suggest the most suitable and affordable foods and diets to users based on their health status and food preferences.

**Objective:**

The present study aimed to design and evaluate a recommender system to suggest healthy and affordable meals and provide a tele-nutrition consulting service.

**Methods:**

This applied three-phase study was conducted in 2020. In the first stage, the food items’ daily prices were extracted from credible sources, and accordingly, meals were placed in three price categories. After conducting a systematic review of similar systems, the requirements and data elements were specified and confirmed by 10 nutritionists and 10 health information management and medical informatics experts. In the second phase, the software was designed and developed based on the findings. In the third phase, system usability was evaluated by four experts based on Nielsen’s heuristic evaluation.

**Results:**

Initially, 72 meals complying with nutritional principles were placed in three price categories. Following a literature review and expert survey, 31 data elements were specified for the system, and the experts confirmed system requirements. Based on the information collected in the previous stage, the Web-based software *TanSa* in the Persian language was designed, developed, and presented on a unique domain. During the evaluation, the mean severity of the problems associated with Nielsen’s 10 principles was 1.2, which is regarded as minor.

**Conclusion:**

To promote food security, the designed system recommends healthy, nutritional, and affordable meals to individuals and households based on user characteristics.

## Introduction

###  Nutritional Challenges, Especially Food Security Concerns

 Malnutrition is defined as a shortage, excess, or imbalance in one’s energy/nutrient intake. This term covers three categories: (1) undernutrition, including wasting (low weight for height), stunting (low height for age), and underweight (low weight for age); (2) micronutrient-related malnutrition, including the shortage of some micronutrients (important minerals and vitamins); and (3) overweight, obesity, and diet-related non-communicable diseases, e.g. cardiac diseases, heart attack, diabetes, and some cancers.^1-3^

 Based on the World Health Organization (WHO) report, a large percentage of the global population suffer from malnutrition; in 2021, about 1.9 billion adults were overweight or obese, while 462 million people were underweight. On the global level, in 2020, 149 million children under the age of 5 years were estimated to be stunted (very short for age), 45 million wasted (too thin for height), and 38.9 million overweight or obese.^1,4^ Like other regions, a considerable percentage of the population in Iran suffer from various forms of malnutrition. Currently, 60% of Iranian adults are overweight or obese^5-7^; 14 Iranian provinces have a relatively insecure to very insecure food status^5,6,8^; more than 10% of Iranian children have moderate-to-high weight loss, and more than 15% have a below-average height.^9^

 Food security refers to the population’s physical and economic access to sufficient, healthy, and nutritional foods to meet nutritional needs and have an active and healthy life.^10^ In addition to physical health impacts, food insecurity can have social and psychological consequences, and this is the reason governmental socioeconomic development programs aim to ensure food security in society.^11^ Measures to guarantee food security for the entire population can be partially implemented through correct programming to provide healthy and affordable diets and nutrition consultation.^12^ Recently, the war between Russia and Ukraine has raised serious concerns about global food security. This concern has been mentioned in many studies in 2022.^13-16^ Also, in Iran, the threat to food security as a result of economic vulnerability has increased in recent years, especially after the re-imposition of US sanctions against Iran. As a result of the present status of food prices and incomes, following a healthy diet has become harder for most Iranian people.^17^

 With the growth of information technologies in recent years, the use of such tools has also expanded in the healthcare system, and patients’ demand to use smart digital device apps has increased sharply.^18-21^ In the domain of nutrition, information technology-based tools have found extensive applications and are utilized to provide healthy and affordable diets and nutrition consultation.^22-24^ The recommender system is one such frequently used nutritional information system.^12^

###  Nutrition Informatics

 Nutrition recommender systems are major technologies allied with nutrition informatics.^12^ They provide an effective tool to support users in modifying their eating behavior and choosing healthier food items.^12,23,25^ These systems recommend foods based on the users’ preferences and principles of a healthy diet; in addition, they can track the clients’ eating behavior, detect health-related problems, and modify their behavior.^12,26,27^

 Different types of recommender systems focus on users’ food preferences and nutritional needs, a balance between user preferences and nutritional needs, and a nutritional recommender system for all groups.^12,25-30^

 In a web-based and electronic environment, this tool can help users find information, services, and items based on their preferences and needs.^31^ Recommender systems can discover users’ interests and predict their priorities, refine the items that are likely to be preferred by users from a large volume of data and information, and recommend the most appropriate ones.^32^ By storing and analyzing the users’ past behaviors, these systems can recommend services and information the users had neglected before but would probably like or need.^31,33^ These systems are frequently utilized to recommend products and services (e.g. movies, books, digital cameras, and financial services) that can best serve user needs and preference.^31-34^

 Recommender systems present an effective technology for the extraction and delivery of valuable information.^34^ Such systems can predict the users’ preferences for unrated food items and suggest new items accordingly.^20,31,34,35^ They have certain technical requirements and require a suitable design based on their types and functionalities.^33,34^ Motivated by the advantages of these systems, recommendation approaches have greatly evolved, and the systems have been incorporated into different disciplines.^31,34^ Several techniques have been put forward for the design of personalized recommendations; this variety in design and techniques has led to different system types, e.g. content-based (CB), collaborative filtering (CF), knowledge-based (KBS), and hybrid recommender systems (HRS).^34,36,37^

 Recently, recommender systems have been employed in the domain of healthy nutrition as a potential solution to help users deal with a large volume of nutrition-related data, including data on foods, nutritional values, healthy foods, recipes, etc.^12,36^ As a result, various studies have been recently conducted on the design and application of recommender systems for nutrition.^12,25-30,36-47^

 In 2017, a nutrition recommender system was designed in Italy. Relying on specialists’ prescriptions, user profiles, and preferences, the system helps users select more appropriate meals and offers healthy and personalized meals.^30^ In a 2018 study in Taiwan, a smart clinical nutrition recommender system was designed for a hospital. The implemented system greatly promoted the quality of nutritional interventions by standardizing clinical nutrition processes and through close monitoring.^29^ A 2015 study in India designed a diet recommender system for patients with diabetes. This system suggested a personalized diet meeting these patients’ needs based on their age, sex, height, weight, physical activity, disease history, and information.^43^ In 2014, a tele-nutrition system was designed in Hungary. This system helped users better monitor their nutritional behaviors, habits, and physical activities, and modify their lifestyle if necessary.^48^

 In previously developed recommender systems, the developers emphasized the recommendation of meals, diets, and nutritional suggestions to ensure the users’ health. In Iran, however, food security should also be taken into account in addition to these issues. Due to nutritional challenges and the sharp rise in food inflation in Iran in recent years (following the US sanctions), reduced access to food and increased malnutrition are inevitable.^49^ This necessitates the provision of a healthy and affordable diet and free and accessible nutrition consultation based on information technology.

## Objectives

 The present study aimed to design and implement a nutrition recommender system to suggest healthy and affordable meals to individuals and families based on their health and economic status. The researchers also attempted to provide a tele-nutrition consulting system based on information technology.

## Materials and Methods

**Figure 1:**
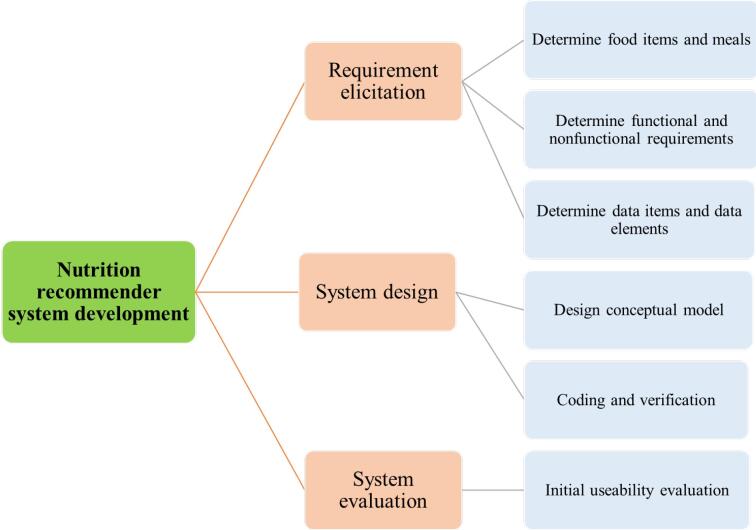
Main Phases of the Study and the Corresponding Steps

 This developmental study was conducted in 2020. The study was conducted in three main phases including requirement elicitation, system design, and system evaluation (Figure 1).

###  Requirement Elicitation

 To determine the system requirements and supervise this process, an expert panel was formed comprising two nutritionists with at least three years of work experience in the field, four health information management experts with at least three years of work experience as a faculty member, a clinician, and a health economic expert with at least three years of work experience as a faculty member.

####  Determining Food Items and Meals

 First, a list of food items was extracted from the Iranian standard Food Frequency Questionnaire (FFQ),^50^ including 168 food items in seven categories (cereals, proteins, dairy, fruits, vegetables, lipids, and junk food). Then, in September 2019, the minimum price of the 168 food items was extracted from different reputable sources including Fruit and Vegetable Fields Organization of Tehran Municipality (for main food items), *Torob* and *Digikala* (for a limited number of special food items that were not available in the list of the fruit and vegetable fields organization of Tehran municipality). Afterward, we used SPSS version 26 to perform a Kolmogorov-Smirnov test in order to assess the distribution of food prices across seven categories. The test results indicated that six of the groups (excluding fruits) exhibited a normal distribution. Subsequently, the mean price and then the standard deviation (SD) of each food item category were calculated by Microsoft Excel 2017. Next, the mean and 2SD were once summed and once subtracted (mean ± 2 SD). The two resulting values showed the lower and upper bounds of the prices, respectively. Finally, prices less than the lower bound, between the lower and upper bounds, and higher than the upper bound were classified as inexpensive, average-price, and expensive food items, respectively. The items were then specifically examined by the nutritionists to determine 72 healthy Iranian meals for the main meal (e.g. lunch) in three price categories (inexpensive, average-price, expensive) based on scientific nutritional principles, e.g. nutritional value, consuming all food groups, and consuming adequate micronutrients. These 72 Iranian meals, as the extracted knowledge, formed the knowledge base of the recommender system.^51^

####  Determining Functional and Nonfunctional Requirements

 Next, through a systematic review based on Valdez’s framework,^52^ the technical^12^ (already published) and methodological aspects of nutrition recommender systems were reviewed, and the data elements and functional and non-functional system requirements were identified.

####  Determining Data Items and Data Elements

 To determine data items, data elements and functional and non-functional system requirements, a questionnaire was developed and presented to 10 nutritionists and 10 health information management and medical informatics experts (all of whom had PhD degrees and were faculty members at Tehran University of Medical Sciences or Shahid Beheshti University of Medical Sciences) according to the Delphi technique. The data items and data elements of the software achieved expert consensus in the first Delphi round.

###  System Design

 The system was designed and developed using the agile methodology and Unified Marked up Language (UML) modeling. To this end, the information flow, functional characteristics, and physical architecture of the system were determined in several meetings with the research team. Then, based on the results of the previous phases, a conceptual model was plotted using Enterprise Architect 12. To design and develop the healthy and affordable meal recommender and tele-nutrition consulting system, PHP, HTML5, and JavaScript Web-based programming languages were used. Moreover, AJAX technologies, CSS package, and Bootstrap 4 were utilized to create the system. All the system coding processes were performed in the Notepad + + environment. The system database was also created using Microsoft SQL Server 2017 in 15 tables. To connect the tables of the database and PHP codes, the PHPmyAdmin interface was used. The Persian (Farsi) system under the commercial name of *TanSa* was executed on a Web-based server (host) and a specific domain responsively.

###  System Evaluation

 System evaluation is an applied research that was conducted based on heuristic evaluation. Heuristic evaluation is a major type of system usability evaluation performed by trained specialists. A well-known method for metaheuristic exploration is Nielsen’s method which comprises 10 principles: (1) Visibility of system status, (2) Match between system and the real world, (3) User control and freedom, (4) Consistency and standards, (5) Error prevention, (6) Recognition rather than recall, (7) Flexibility and efficiency of use, (8) Aesthetic and minimalist design, (9) Help users recognize, diagnose, and recover from errors, (10) Help and documentation. These 10 principles help identify the possible problems of the system in heuristic evaluation.^53^

 In this phase, preliminary software evaluation was performed by four evaluators, including two medical informatics and two health information management experts familiar with Nielsen’s evaluation. The experts were asked to identify the problems based on the 10 principles and for each task and functionality designed for the system. Finally, the number of problems was obtained, and their mean severity was calculated with SPSS and reported.

## Results

###  Requirement Elicitation

####  Food items and meals

 Initially, the food items were categorized by the expert panel. To this end, through in-person visits and a review of electronic sources from credible organizations and references, the prices of 168 food items were collected based on the Iranian FFQ. Then, by calculating the mean and SD of the prices, the 168 food items were classified as inexpensive, average-price, or expensive. The FFQ classifies food items into seven categories of cereals, proteins, dairy, fruits, vegetables, lipids, and junk foods. We excluded fruit items from the analysis as they did not exhibit a normal distribution, and their inclusion was not crucial for determining lunch meals. The same classification was adopted in this study (Table 1).

**Table 1:** Classification of Food Items Group Based on Price Rate

**Food Items Group**	**Food Items Number Based on Price Rate**		
	**Expensive**	**At an Average Price**	**Inexpensive**
Cereals	6	9	6
Eggs, meat, and legumes	7	6	12
Dairy	4	5	7
Vegetables	4	5	9
Lipid	4	5	6
Junk Food	7	11	10

 Next, in the expert panel, 72 meals were presented based on their nutritional value and daily price in three categories inexpensive (24 meals), average-price (19 meals), and expensive (29 meals), which constituted the knowledge base of the recommender system.

####  Functional and Non-functional Requirements

**Figure 2:**
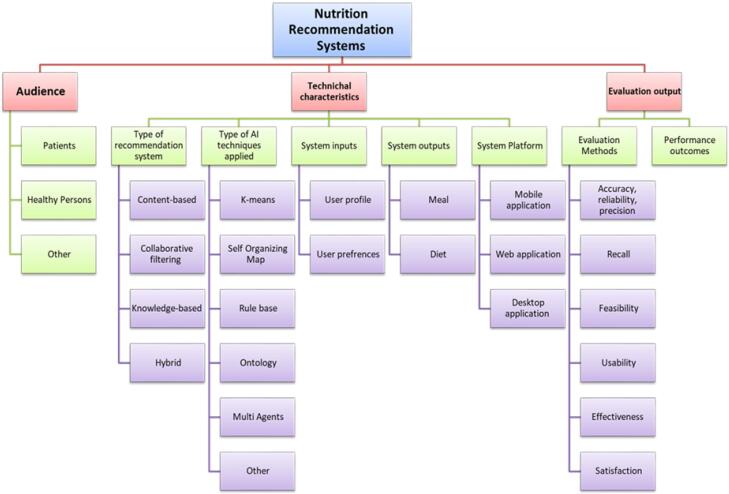
Assessing the Three Main Aspects of Nutrition Recommender Systems

 Next, a systematic review was conducted on relevant studies. The main results related to technical aspects were published before.^12^ The most interesting findings concerning the methodological aspects of nutrition recommender systems are categorized and summarized in Figure 2.

####  Data Items and Data Elements

 The results of the systematic phase and the expert panel sessions, as well as all the data elements of the recommender and tele-nutrition consulting system, were incorporated into a questionnaire that was presented to experts based on the Delphi technique (results presented in Tables 2 and 3).

**Table 2:** Recommender System Data Items from the Experts’ Point of View

**Row**	**Data Items**	**Mean**	**Necessity**
1	Gender	4.25	✓
2	Age	4.50	✓
3	amount of money that the user can allocate per month to his/her nutrition	4.55	✓
4	Pregnancy status	4.75	✓
5	Breastfeeding status	4.75	✓
6	food allergies	4.90	✓
7	Foods or meals that the user does not like	4.20	✓

**Table 3:** Data Elements of the Tele-nutrition Consulting System According to the Experts

**Row**	**Data Elements**		**Mean**	**Necessity**
1	Demographic data	Name	2.70	Confirmed/essential
2		Gender	4.65	Confirmed/essential
3		Age	4.70	Confirmed/essential
4		National code	2.70	Confirmed/essential
5		Nationality	3.15	Confirmed/essential
6		Ethnicity	3.40	Confirmed/essential
7		Religion	2.90	Confirmed/essential
8		Province	3.30	Confirmed/essential
9		City	3.20	Confirmed/essential
10		Email	2.80	Confirmed/essential
11		Phone No.	3.45	Confirmed/essential
12	Scio-economic data	Marital status	3.75	Confirmed/essential
13		Job	4.35	Confirmed/essential
14		Level of Education	4.00	Confirmed/essential
15		Amount of money that the user can allocate per month to his/her nutrition	4.60	Confirmed/essential
16	Personal health status data	Height	4.50	Confirmed/essential
17		Weight	4.70	Confirmed/essential
18		Physical activity rate and type of activity	4.80	Confirmed/essential
19		Pregnancy status	4.85	Confirmed/essential
20		Breastfeeding status	4.85	Confirmed/essential
21		Food allergies	4.75	Confirmed/essential
22		Wrist size	3.70	Confirmed/essential
23		Waist size	4.50	Confirmed/essential
24		Disease status (diabetes, hypertension, hypothyroidism, hyperlipidemia, fatty liver, kidney disease, heart disease, and gastrointestinal disease)	4.90	Confirmed/essential
25		Blood type	2.40	Removed/non-essential
26		Taking drugs	4.45	Confirmed/essential
27		Dietary supplements	4.25	Confirmed/essential
28	Food preferences	Favorite food and diets	4.10	Confirmed/essential
29		Foods and meals that the user does not like	4.10	Confirmed/essential
30	Eating behavior	24-hour food recall questionnaire	4.60	Confirmed/essential

 The experts deemed all the suggested data elements as necessary (Table 2).

 Based on Table 3, from 30 suggested data, 25 elements achieved a high mean score and were thus regarded as essential. Four data elements (name, national ID number, religion, email address) that achieved a lower mean were still deemed essential data elements. Nevertheless, blood type (with a mean value of 2.4 out of 5) was deemed unnecessary and eventually removed.

 As the recommender system was knowledge-based and extracted rules based on a rule base, a set of rules was defined (Table 4).

**Table 4:** Rules for the Knowledge Base of the Recommender System

**Scope of Rules**	**Rules**
Rules for age	IF the user's age is 8-14 years, THEN recommend meals X_1_ to X_n_. IF the user's age is 14-18 years, THEN recommend meals X_1_ to X_n_. IF the user's age is 18-60 years, THEN recommend meals X_1_ to X_n_. IF the user's age is > 60 years, THEN recommend meals X_1_ to X_n_.
Rules for economic status^*^	IF the user can spend < 5000000 Iranian rials a month on nutrition, THEN recommend inexpensive meals. IF the user can spend 5000000-10000000 Iranian rials a month on nutrition, THEN recommend inexpensive and average-price meals. IF the user can spend > 10000000 Iranian rials a month on nutrition, THEN recommend inexpensive, average-price, and expensive meals.
Rules for pregnancy status	IF the user is pregnant, THEN remove Z_1_-Z_n_ meals from the recommendations.
Rules for breastfeeding status	IF the user is breastfeeding, THEN remove T_1_-T_n_ meals from the recommendations.
Rules for food allergies	IF the user is allergic to I_1_-I_n_ meals, THEN remove these meals from the recommendations.
Rules for food preferences	IF the user does not prefer J_1_-J_n_ meals, THEN remove these meals from the recommendations.

*The limit of 500,000 Iranian rials was determined based on the inflation of Iran in September 2019 and should be updated in the future.

###  System Design

**Figure 3:**
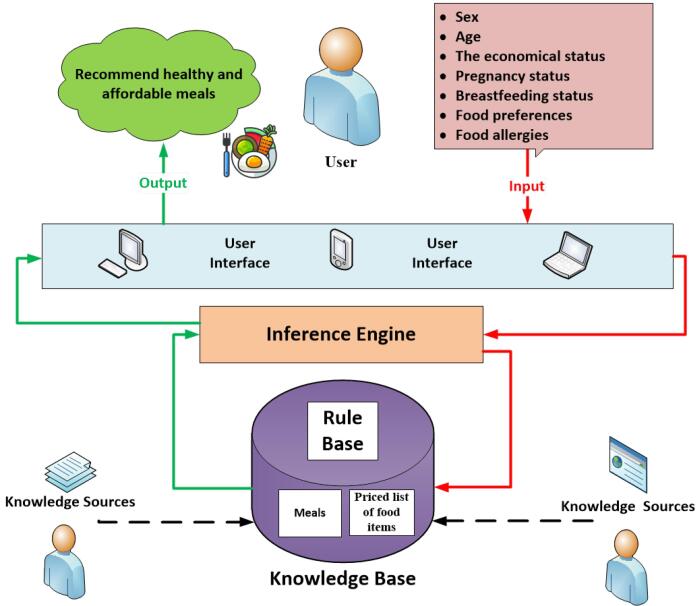
Overall Schematic Representation of the Nutrition Recommender System Architecture.

**Figure 4:**
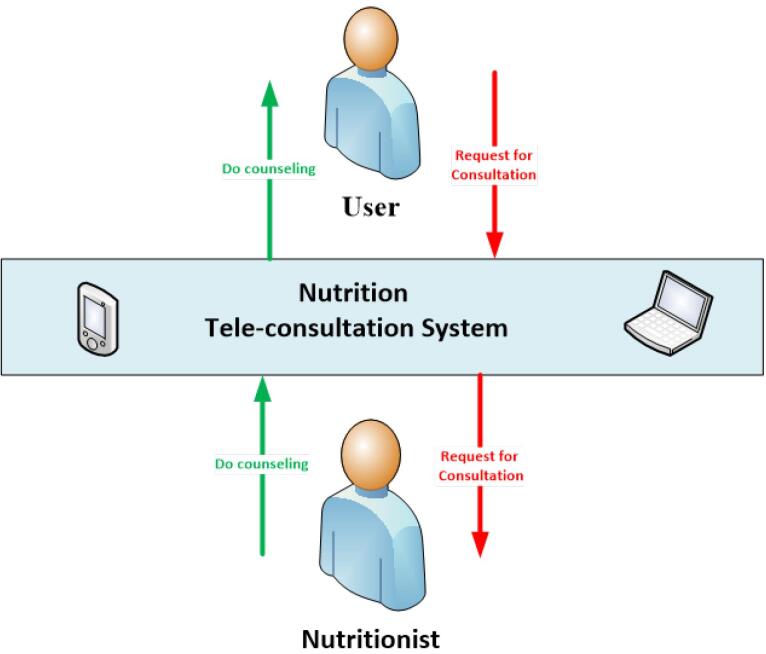
Overall Schematic Representation of the Tele-nutrition Consulting System Architecture

 After eliciting the requirements and needs, the software was developed. Figures 3 and 4 respectively display the overall system architecture, the overall architecture of the teleconsultation part, and the home page. Since the software was designed for the Persian language, the images were translated into English for better display.

 In this system, actors include the main user, nutritionist, nutritional administrator, economic administrator and technical administrator (system manager).

 The end user (applicant): This user is the main client of the *TanSa* system who wants to receive the two main services of the system, that is, to receive healthy and affordable meals from the recommender system (at the individual and household level) and also to receive nutritional advice and diets from nutritionists.

 Nutritionist: This user is responsible for providing remote nutritional advice or prescribing a diet to the applicant.

 Nutritional admin: This user is at a higher level than the nutritionist. The responsibility of this user is to supervise at a higher level all nutritional functions of the system and they can supervise the interaction of applicants with the system and applicants with nutritionists. This user is responsible for assigning a nutritionist to the requests sent by the applicants. This user can receive various reports based on the information entered in the system for better management and monitoring.

 Technical admin: This user is responsible for all aspects of the system’s technical performance, such as user management, system security, database management, etc.

 Economic admin: This user is responsible for calculating and classifying food items in terms of price according to economic principles and updating food prices.

**Figure 5:**
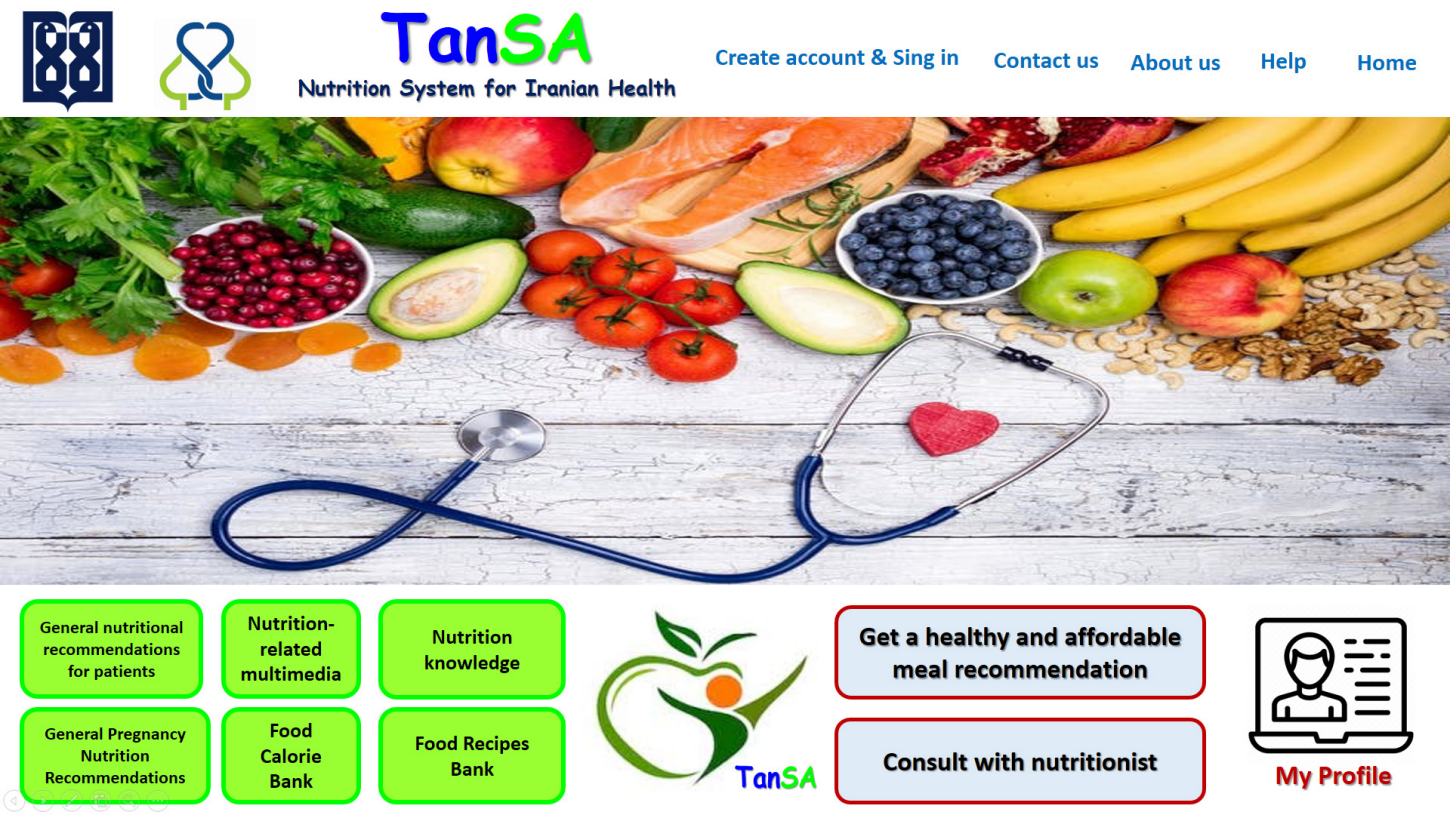
System Home Page

**Figure 6:**
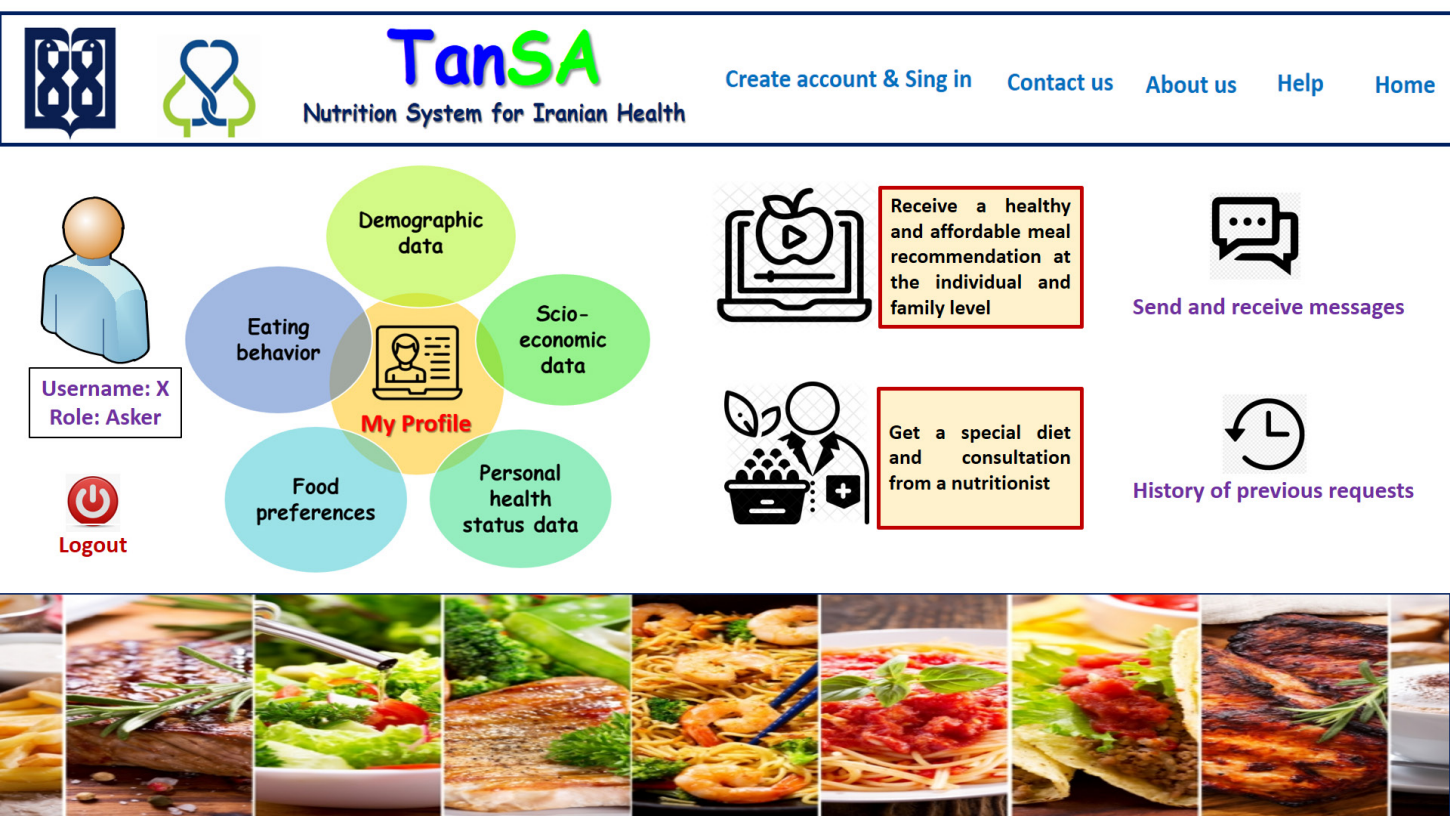
System Asker Role Page

**Figure 7:**
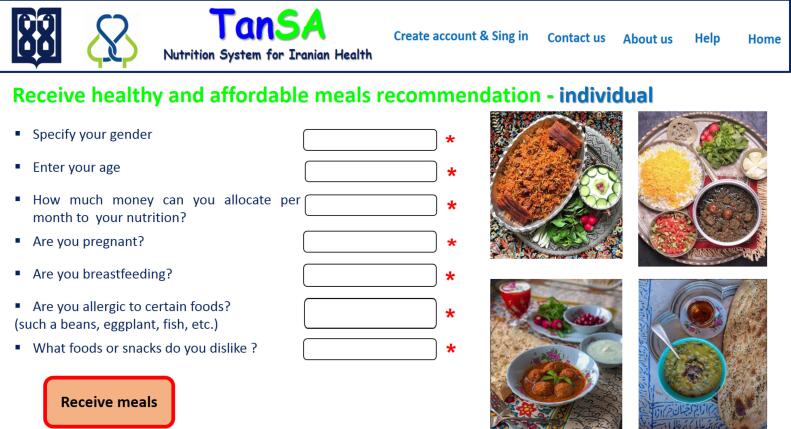
Input Page of Nutrition Recommender System.

**Figure 8:**
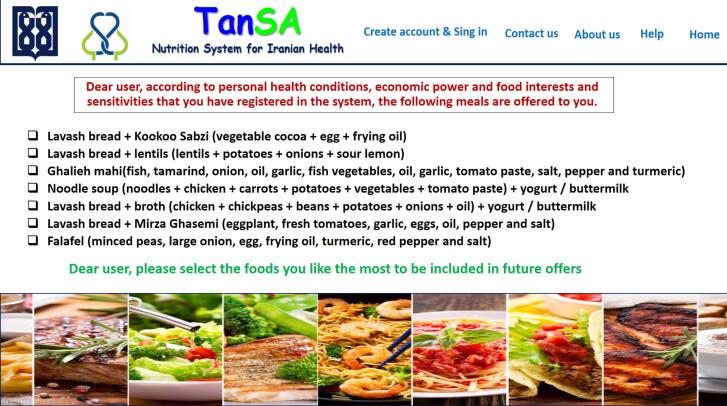
Output Page of the Nutrition Recommender System

 In addition, Figures 5‒8 depict the user interface of some pages of *TanSa*.

###  System Evaluation

 After developing the system and its online execution, it was evaluated by four experts familiar with metaheuristic evaluation. In terms of the visibility of system status, three problems (12%) with a mean severity of 1 were identified. “The nutrition admin cannot receive and save user reports as pdf and Excel files” (one evaluator), “using non-Persian words” (one evaluator), and “an inactive link on the home page” (one evaluator) were mentioned.

 As for the match between the system and the real world, five problems (20%) with a mean severity of 1.2 were identified. “Left alignment of Persian search boxes” (two evaluators), problems with the “credibility of the sources used on the home page” (one evaluator), and “using a far-fetched language” (one evaluator) were reported.

 In terms of consistency and standards, two problems (8%) with a mean severity of 1.5 were identified. “Different header colors across pages” (one evaluator) and “different locations of the back icon across pages” (one evaluator) were noted.

 In terms of aesthetic and minimal design, four problems (16%) with a mean severity of 1.2 were identified. Problems with “inappropriate design of the data entry form in user profile” (two evaluators) and “unappealing and large icons” (two evaluators) were noted.

 As for error prevention, five problems (20%) with a mean severity of 2 were identified. “Not displaying an error message after entering too many characters in the field” (one evaluator), “not displaying an error message after entering characters in a forbidden language” (one evaluator), “not displaying an error message after entering incomplete information” (one evaluator), and “inappropriate information classification for later user visits” (one evaluator) were mentioned.

 In terms of recognition rather than recall, two problems (8%) with a mean severity of 1.5 were identified. Two evaluators reported “unmarked mandatory fields”.

 As for user control and freedom, two problems (8%) with a mean severity of 1.5 were identified. Two evaluators noted the “absence of a site map”.

 In terms of help and documentation, two problems (8%) with a mean severity of 1 were identified. “Complicated and difficult explanations” (one evaluator) and “not using images for training how to use the system” (one evaluator) were noted.

 The evaluators reported no problems in the domains of “flexibility and efficiency of use” and “help users recognize, diagnose, and recover from errors”. Table 5 shows the results of the system evaluation.

**Table 5:** Problems Identified in the Recommended System Based on the Heuristic Evaluation

**Row**	**Principles of Nielson Heuristic Evaluation**	**Total Number of Problems**	**Percentage**	**Mean Severity**	**Problem Severity**
1	Visibility of system status	3	12	1	Minor problem
2	Match between the system and the real world	5	20	1.2	Minor problem
3	Consistency and standards	2	8	1.5	Minor problem
4	Aesthetic and minimalist design	4	16	1.2	Minor problem
5	Error prevention	5	20	2	Small problem
6	Recognition rather than recall	2	8	1.5	Minor problem
7	User control and freedom	2	8	1.5	Minor problem
8	Flexibility and efficiency of use	0	0	0	No problem
9	Help users recognize, diagnose, and recover from errors	0	0	0	No problem
10	Help and Documentation	2	8	1	Minor problem
**Total**		**25**	**100**	**1.09**	Minor problem

## Discussion

###  Principal Results

 Economic factors greatly contribute to people’s purchasing power and consumption of healthy foods. Furthermore, due to the significance of food security, the present study developed a nutrition recommender system to suggest healthy meals based on individuals’ and families’ economic status. The household economic status and adequate nutrition literacy for the selection and purchase of food items are the main factors affecting food security, and the variable of the price should also be considered as a major influential factor.^6,8,54^

 The finding suggested the effect of economic factors on food security, which confirms the approach and objectives of the present study. Numerous nutrition recommender systems have been designed and implemented worldwide, but they solely focus on users’ nutritional needs and preferences and ignore economic factors when recommending meals or diets.^12,25-30,36-47^ Due to the contribution of economic factors to food security, and due to the recent inflation in food prices in Iran, the present study designed a nutrition recommender system by taking economic factors into account.

 The outputs of nutrition recommender systems are mostly divided into three categories of diets, meals, and recommendations. There is still no consensus on the use of smart systems for recommending personalized diets; moreover, as this study aimed to promote food security, meal recommendation was regarded as the output of the recommender system, which is also the case in many other studies.^12,25-30,36-47^

 After eliciting system requirements, based on the agile approach, the Web-based *TanSa* system was designed. This system provides two principal services “recommending healthy and affordable meals for individuals and households” and “offering a tele-nutrition consulting system”. For the first service, a nutrition recommender system was designed which offers healthy and affordable meals based on the Iranian cuisine to meet the nutritional needs of individuals and households based on seven items (sex, age, economic status, pregnancy status, breastfeeding status, food preferences, and food allergies). For the second service, individuals can receive specialized teleconsultation and diets from nutritionists based on their profiles and nutritional records completed on the systems. Moreover, a nutritional admin was defined for the system to supervise all the nutritional aspects of the system (e.g. changing and adding meals, the quality of consultations provided by nutritionists) and receive different reports for decision- and policy-making, if necessary.

 Another user called “economic admin” was also designed to supervise the economic aspects of the recommender system (e.g. calculation and pricing of food items and meals) and modify the system if necessary. It is suggested that a team comprising health economy and management experts take charge of this part of the system. A technical admin was also defined to supervise and manage all the technical processes, e.g. registration and system and database security.

 Bianchini et al designed a hybrid Web-based meal recommender system for healthy individuals using Java language. The researchers used the SQL Server to design the database. As the data analysis and inferencing technique was ontology-based, the principal module in the architecture of this system belonged to the ontology analysis engine. The present study is similar to the aforementioned study in numerous aspects, e.g. the type of the recommender system, the Web-based platform, using Java coding language, and designing the database by SQL Server. In the study by Bianchini et al, the data inferencing method relied on ontology-based algorithms, while in the present study, information inferencing relied on a network of rules.^30^

 Another study developed a content-based and web-based recommender system coded using PHP, HTML5, and JavaScript languages. This study used the SQL Server for designing and managing the database. The present study was similar to Agapito’s study in terms of designing a web-based platform, and system architecture, using PHP, HTML5, and SCRIPT coding languages, and designing the database with SQL Server.^26,55^

 Theoretically, the evaluation of health informatics applications can improve the quality of care, reduce costs, and control the safety and effectiveness of health information systems (HIS).^56^ Evaluation should be performed to promote the performance of health informatics applications by applying prior experience to recognize more effective techniques or methods, explore failures, and learn from faults.^57^ Most nutrition recommender systems are in earlier phases of research (first to the third levels of system research mentioned in the five-stage model for comprehensive research on health informatics projects).^58^

 Heuristic evaluation is a simple and quick method for system usability evaluation that is performed by experts, not users. Nielsen recommended three to five evaluators for this method. If fewer than four people do the evaluation, the results cannot be reliable; if more than five people conduct the evaluation, the number of identified problems will be higher but the cost-effectiveness of the evaluation will not be optimal.^55,59-61^

 In the present study, four experts evaluated the system. Other studies also asked three to four evaluators for evaluation.^62,63^ The evaluators identified 25 problems, the majority of which belonged to “error prevention” and “match between system and the real world” principles. In the study by Ahmadian et al, 53 problems were identified for a radiology information system, the majority of which belonged to the same two principles.^62^ In the study by Khajouei et al, 163 errors were identified in the emergency department information system, most of which belonged to “error prevention”.^63^ The results of both studies are consistent with the findings of the present study.

 In a study by Bozkurt et al, using the think-aloud protocol as another usability evaluation method, the researchers evaluated a nutritional self-care system. The opinions of 10 system users who participated voluntarily were acquired through semi-structured interviews. The results revealed that the system usability score for nutrition and nutrition education was 77%. According to the users, the system had problems with credible content and easy navigation that were identified through evaluation.^64^ The main difference between this and the present study lies in the method of usability evaluation. In the mentioned study, the researchers employed the think-aloud protocol and based the evaluation on the users’ opinions. Nevertheless, in the present study, the usability evaluation method was Nielsen’s heuristic evaluation based on experts’ opinions. The problems with the “credibility of sources and contents” and the “absence of a site map” in the present study correspond to the problems with “content” and “system navigation” in the study by Bozkurt et al,^64^ respectively.

## Limitations

 One potential limitation of this study is that the prices of all 168 food items were not available in a single reference source. To overcome this challenge, the researcher used three different sources and organizations to determine the prices. While this approach allowed for a comprehensive analysis, there may have been variations in the pricing data that could have influenced the accuracy of the results. Additionally, due to the study’s focus on the Iranian context, the findings may not be generalizable to other regions or populations with different cultural and economic backgrounds. In fact, this system is designed for Iranian people. Furthermore, it should be noted that this study did not evaluate the long-term impact of the nutrition recommender system on the health status of its users.

## Comparison with Prior Works

 Numerous nutrition recommendation systems have been developed worldwide, with a primary focus on medical and health-oriented aspects. However, economic considerations have been overlooked in all of these systems, highlighting the need for innovative approaches to address affordability barriers to healthy eating. In this context, our research represents a significant contribution as it is the first study worldwide to recommend healthy and affordable meals to users, considering both their economic status and health status. By incorporating economic considerations into our recommendations, we developed a novel approach to promote food security and facilitate access to nutritious food options, particularly for low-income groups.

## Recommendations for Future Research

 Further studies are needed to evaluate the impact of nutrition recommender systems such as *TanSa* on the management of chronic diseases, public health, and other health issues. These studies can assess outcomes such as food security, weight management, nutrient delivery in clinical settings. By exploring the effectiveness of these systems on health outcomes, researchers can optimize their design and implementation and improve healthcare services’ equity and accessibility. Additionally, future studies can explore the potential of these systems to promote healthy behaviors and prevent chronic diseases, highlighting their potential to revolutionize healthcare technology and improve public health outcomes.

## Conclusion

 In conclusion, the findings of this study and others suggest that recommender systems have a significant potential for use in various healthcare domains, including nutrition. By suggesting dietary plans, meals, and nutritional recommendations, a nutrition recommender system (a technology belonging to nutrition informatics) can assist users in selecting healthier foods, improving their overall lifestyle, and promoting good health. One notable advantage of such a system is its ability to consider both the health and value of food, as well as the associated costs based on an individual or family’s income level. Given the economic crises and high inflation experienced in Iran in recent years, employing this system could help improve food security within the community. The current study sought to design and develop a recommender system that recommends affordable and nutritious meals, with a focus on enhancing food security among low-income groups. Through the use of information and communication technology, the study created a platform that provides personalized nutritional tele-consultation by experts, thereby increasing access to healthcare services and promoting equity. The evaluation of the *TanSa* Persian nutrition recommendation system revealed its excellent performance and the potential to serve as an example for other countries aiming to improve food security, particularly following the food security concerns arising from the Russia-Ukraine war. Overall, this study highlights the value of recommender systems in improving healthcare outcomes and addressing health challenges, such as food insecurity, through innovative technology.
